# Irritable Stomach

**Published:** 1854-01

**Authors:** 


*Irritable Stomach.*— Dr. Robertson recommends "painfully flexing the little finger of the patient, to compel his irritable stomach to retain medicine." He says they cannot "throw up the medicine" while the painful flexure is maintained. Dr. T. S. Bell uses a tumbler, applied as a dry cup over the stomach. The N. O. Med. and Surg. Journal recommends good champagne wine and ice. Dr. Bowling, of the Nashville Journal, considers *frozen* champagne as *the* remedy; he says, "in the case of a sound stomach, we feel authorized to say, that one that would reject ice-champagne, under any circumstances, might be set down as unreasonably fastidious and refractory."—*From the N. H. Journal of Medicine.*

